# Mutagenesis of Intrinsically Disordered Domain Impacts Topoisomerase IIα Catalytic Activity

**DOI:** 10.3390/ijms26083604

**Published:** 2025-04-11

**Authors:** Jeong Won Chang, Addison K. O’Brian, Allison J. Thomas, Mattalyn R. Hardin, Brooke D. Latham, Daniel Ngabonziza, Lily G. Simpson, Benjamin D. Wade, Laura Kühnhenrich, Nina M. Thompson, Clark E. Endsley, Joseph E. Deweese

**Affiliations:** 1 Biological, Physical, and Human Sciences Department, Freed-Hardeman University, Henderson, TN 38340, USA; 2 Department of Biochemistry, Vanderbilt University School of Medicine, Nashville, TN 37235, USA

**Keywords:** topoisomerase IIα, TOP2A, intrinsically disordered domain, protein disorder, carboxy-terminal domain, DNA, relaxation, cleavage, decatenation, PSICalc

## Abstract

Human topoisomerase IIα and IIβ regulate DNA topology and knots in chromosomes during crucial cellular processes, making these enzymes common targets for anticancer drugs. However, selective inhibition of topoisomerase IIα (TOP2A) is desired to decrease adverse effects, which may be mediated by topoisomerase IIβ (TOP2B). The main region of difference between the two isoforms is the intrinsically disordered C-terminal domain (CTD), which is being studied as a target for selective inhibition. Our previous work examined several regions within the CTD to determine whether those regions impact biochemical function. In this current study, we designed and constructed four TOP2A mutants with amino acid substitutions in the CTD, which were then assessed for impact on biochemical activity. V1482D exhibited increased levels of relaxation, while both V1482D and K1520I exhibited increased levels of decatenation. No major impact on DNA cleavage or binding were observed with any of the mutants. The isolated impact of the changes on relaxation and decatenation supports the concept that the CTD can affect one aspect of the enzyme’s function in an isolated manner, which was seen in our previous study. Taken together, these results suggest that modification of specific positions within the CTD affects substrate selection. These results are mapped onto the CTD for consideration of potential regions to target for inhibition of TOP2A.

## 1. Introduction

Disordered protein domains are a growing theme in protein studies [1,2,3,4]. Protein disorder results from features of the sequence, such as being enriched or depleted in certain amino acids [5]. The result is a lack of regular secondary structural elements that results in a flexibility of the region or protein. Intrinsically disordered proteins (IDP) and intrinsically disordered protein regions (IDR) appear to fold or assume a stable conformation upon binding to partners such as proteins, DNA, or RNA [6,7,8]. This induced folding appears to allow IDPs and IDRs to interact with more than one binding partner, which allows proteins to be multifunctional without requiring separate binding sites for each function [9]. The roles of IDPs and IDRs are diverse and include protein-protein interactions, protein-nucleic acid binding, phase condensation, and regulation of catalytic activity [3,10,11,12,13,14,15].

Studying the roles of such domains can be challenging since they lack a predictable regular secondary structure, which often challenges structural analysis via cryo-electron microscopy and X-ray crystallography [16]. Unique approaches and tools are needed to help complement more traditional approaches. Even computational tools such as AlphaFold yield limited results with unstructured domains [17]. Type IIA DNA topoisomerases found in eukaryotes have an IDR at the carboxy terminus that has been difficult to study [14,16,17,18,19,20,21,22,23,24,25,26,27,28,29,30,31].

DNA topoisomerase II (TOP2) is a type IIA topoisomerase that maintains DNA topology and alleviates knots and tangles in and among chromosomes [32,33,34]. TOP2 regulates the topology of DNA through controlling the level and extent of supercoiling using a double-strand DNA passage mechanism that involves a transient double-stranded DNA break [32,33,34,35]. The enzyme has three gates, N-gate (ATPase/transducer), DNA-gate (TOPRIM/cleavage/ligation domain), and the C-gate (lower region before CTD), that open and close in turn to ensure the enzyme remains intact [36]. The “top” of the enzyme is the N-terminal ATPase domain, followed by a transducer domain that connects with and communicates with the TOPRIM and cleavage/ligation domains within the core of the enzyme [37,38]. There is also a lower region that serves as a lower gate, called the C-gate for the C-terminal gate, before the intrinsically disordered CTD [29].

Human TOP2 is found as two isoforms in cells that are expressed from separate genes: topoisomerase IIα (TOP2A) and topoisomerase IIβ (TOP2B). TOP2A manages supercoiling and topological entanglements during replication and mitosis [32]. TOP2B is involved in regulating DNA topology during transcription and chromatin remodeling [39]. Both isoforms are targeted by widely-used anticancer drugs such as etoposide and doxorubicin [40]. Unfortunately, several adverse events are associated with these therapies, and some of these adverse events may be due to the action of the drug on TOP2B [41,42,43]. Therefore, there is increased interest in finding ways to more selectively target TOP2A, and there have been a few compounds identified that offer some level of selectivity [40].

The intrinsically disordered CTD of TOP2A is over 300 amino acids in length [17,19,24,30]. While this region is not required for catalytic function and appears to be variable among type IIA topoisomerases, it is needed in cellular contexts for localization and regulation of function in a cell cycle-specific manner [18,20,44,45]. This region differs significantly between TOP2A and TOP2B, making it a potential point of differentiation between the two isoforms [22,29,30,40].

Structural and biochemical studies have found that deletion of portions of the CTD can affect DNA substrate selection and the ability to facilitate phase condensation [14,19,20,21,22,30,45]. CTD mutations can impact catalytic function, implying that the CTD is involved in regulating function [30,46]. Our previous studies examined a series of Ser/Thr residues in the CTD to determine whether mutations in purified enzymes impacted biochemical function [30,46]. Several regions were identified that either increased or decreased catalytic activity, which is consistent with other previous research examining the CTD [30].

**Figure 1:**
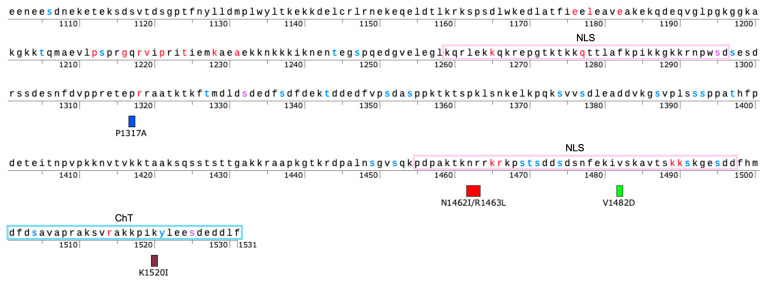
Map of CTD Region of TOP2A with key features and mutant sites. Mutants are denoted by a box as P1317A (blue), N1462I/R1463L (red), V1482D (green), and K1520I (maroon). Nuclear localization signals (NLS, purple box) and Chromatin Tether (ChT, teal box) are denoted. Residues in red represent positions that are invariant among TOP2A [31], blue sites are known to be phosphorylated in association with mitosis [29,48], and purple represents sites that are both invariant and are known to be modified.

To guide our selection of additional CTD sites for evaluation, we utilized PSICalc. PSICalc is a pattern discovery tool that utilizes a derivation of the k-modes algorithm to compare positions within an amino acid sequence and identify interdependencies among positions [17]. Previously, we applied the PSICalc tool to a dataset of 347 TOP2 sequences from various domains of life [17,31,47]. This study identified several amino acid positions within the CTD that are interdependent with amino acids in the N-terminal ATPase domain. We selected and mutated five CTD positions that were identified in this dataset and generated four mutants (one as a double-mutant) as outlined in Supplemental Table S1 and shown in Figure 1. In the time since the generation and initial analysis of the mutants, it was found that the original clusters were anomalous due to an issue with the clustering algorithm, which was corrected and reported in a previous study [31]. Using the corrected algorithm, these positions are found in clusters with other sites [31]. Since the mutant enzymes were made before the algorithm error was discovered, we continued with our analysis of these mutants and report the results in this current study.

In this current work, we describe results with four TOP2A CTD mutants: P1317A, N1462I/R1463L, V1482D, and K1520I. Our results indicate that some mutations have little or no effect on catalytic activity, while others may slightly increase or decrease activity. These results are reported and considered in light of recent bioinformatic and biochemical analyses of the CTD [31].

## 2. Results

### 2.1. Plasmid DNA Relaxation by CTD TOP2A Mutants

**Figure 2:**
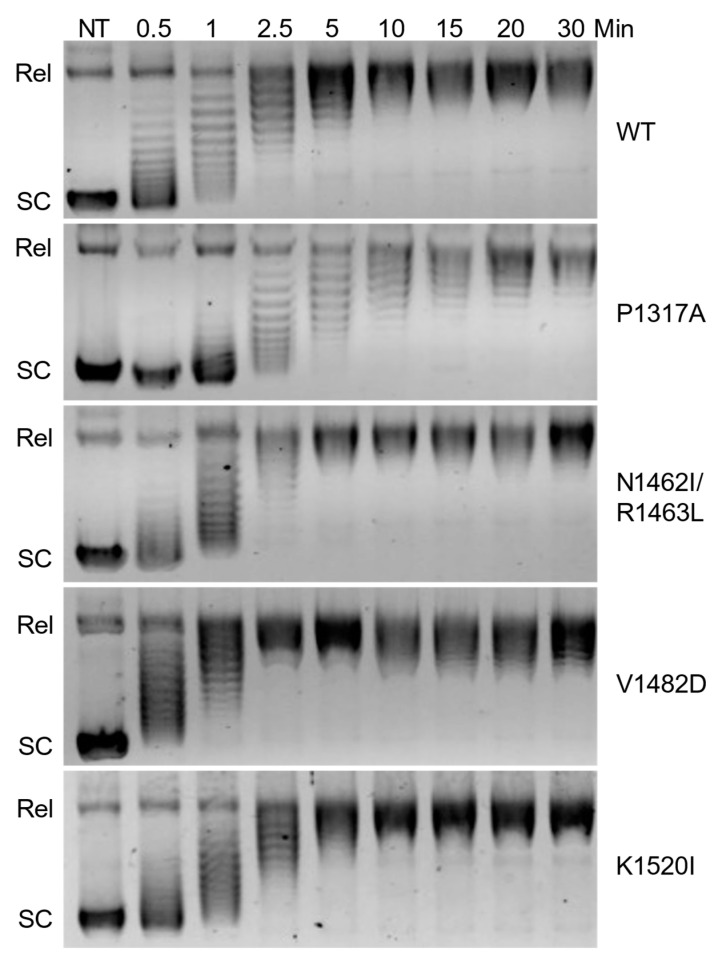
Relaxation time course of DNA by wild-type TOP2A (WT) and mutant TOP2A (P1317A, N1462I/R1463L, V1482D, and K1520I). Representative gel images show the migration of supercoiled plasmid in the absence of TOP2A (NT) as well as in the presence of TOP2A at increasing time points (0.5–30 min). The relaxed plasmid (Rel) migrates more slowly than supercoiled (SC) plasmid. Gel images are representative of three or more experiments.

**Figure 3:**
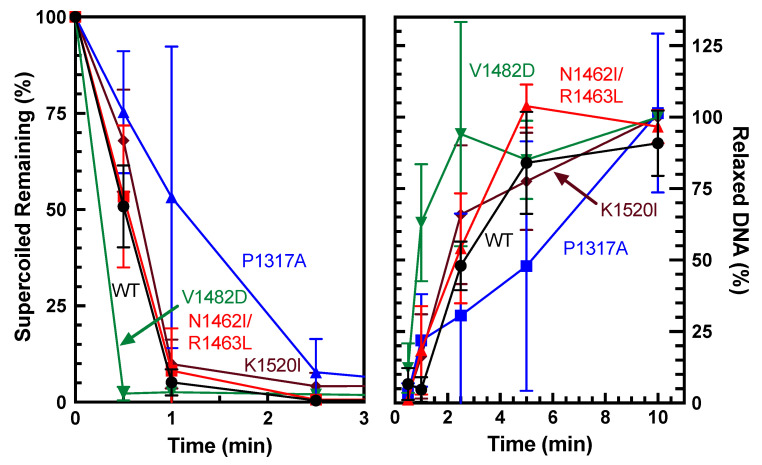
Quantification of plasmid DNA relaxation by WT TOP2A and TOP2A mutants P1317A, N1462I/R1463L, V1482D, and K1520I. Relaxation was quantified both as a percentage of supercoiled remaining (**left**) and gain of relaxed DNA (**right**). Error bars represent the standard deviation of at least three experiments.

Overall catalytic activity is commonly monitored using plasmid-based DNA relaxation assays combined with gel electrophoresis [30]. The ability of TOP2A to relax negatively supercoiled DNA was determined via relaxation assays using pBR322 plasmid. Relaxation capabilities of the different mutants were compared against wild-type (WT) via time courses (Figure 2). Since relaxed DNA migrates much slower than supercoiled DNA in an agarose gel, the first time point at which all the DNA is in line with the relaxed band is the time at which relaxation has been completed. WT enzyme fully relaxed the substrate between 5 and 10 min. As seen in Figure 3, quantification of the loss of supercoiled substrate and the gain of relaxed substrate indicates that V1482D shows increased relaxation activity, while P1317A shows a decrease in activity compared to WT. Thus, each of the mutants is catalytically active, but the comparative activities differ slightly with some of the mutations. Also, V1482D appears to start slightly supercoiling the DNA at the higher time points, which may reflect the higher activity of the enzyme. This effect has been observed previously in another CTD mutant [30] and is also similar to what is seen when higher levels of WT TOP2A are used in a relaxation assay. While relaxation captures overall catalytic activity, additional assays are needed to assess whether portions of the catalytic cycle are affected by these mutations.

### 2.2. Plasmid DNA Cleavage by CTD TOP2A Mutants

**Figure 4:**
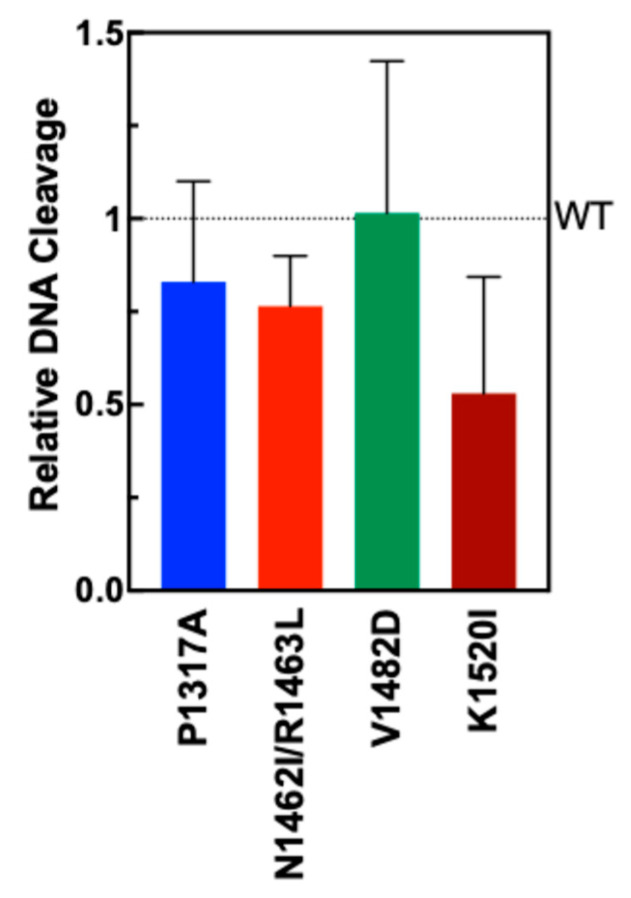
Plasmid DNA cleavage by WT TOP2A and TOP2A mutants P1317A, N1462I/R1463L, V1482D, and K1520I. Quantification represents the linearized plasmid in the gel (double-strand breaks). Error bars represent the standard deviation from four experiments.

To test the extent to which the TOP2A mutants could form double-strand breaks in DNA, plasmid DNA cleavage assays were performed. Negatively supercoiled plasmid (pBR322) was incubated with TOP2A WT and mutant enzymes. The percentage of double-stranded DNA cleavage formed after a 6 min assay is shown in Figure 4 compared with WT (dotted line). Though the results are not statistically significant, mutants P1317A, N1462I, R1463L, and K1520I all show a slight decrease in activity, with the largest decrease shown by mutant K1520I. Mutant V1482D is comparable to wild-type TOP2A in cleavage activity. These results indicate that the mutations do not significantly impact DNA cleavage activity for any of the mutants.

Because TOP2A contains two active sites, it is possible to monitor the level of coordination between them by tracking double-strand breaks (DSBs) and single-strand breaks (SSBs) in a plasmid DNA substrate. Agarose gel electrophoresis separates the nicked (SSB) DNA from the linear (DSB) plasmid DNA. The relative coordination between the active sites of the dimer can be approximated using a DSB/SSB ratio. If the enzyme is perfectly coordinated, a high ratio of DSB compared to SSB would be expected. While evidence indicates that the two active sites can cut independently, there is a degree of coordination between the two halves [49]. In general, the WT enzyme displays more SSB than DSB when reactions are stopped with SDS and no TOP2 poisons are present, making the ratio less than one [30]. This may be a protective feature of the enzyme mechanism to prevent forming too many DSBs.

**Figure 5:**
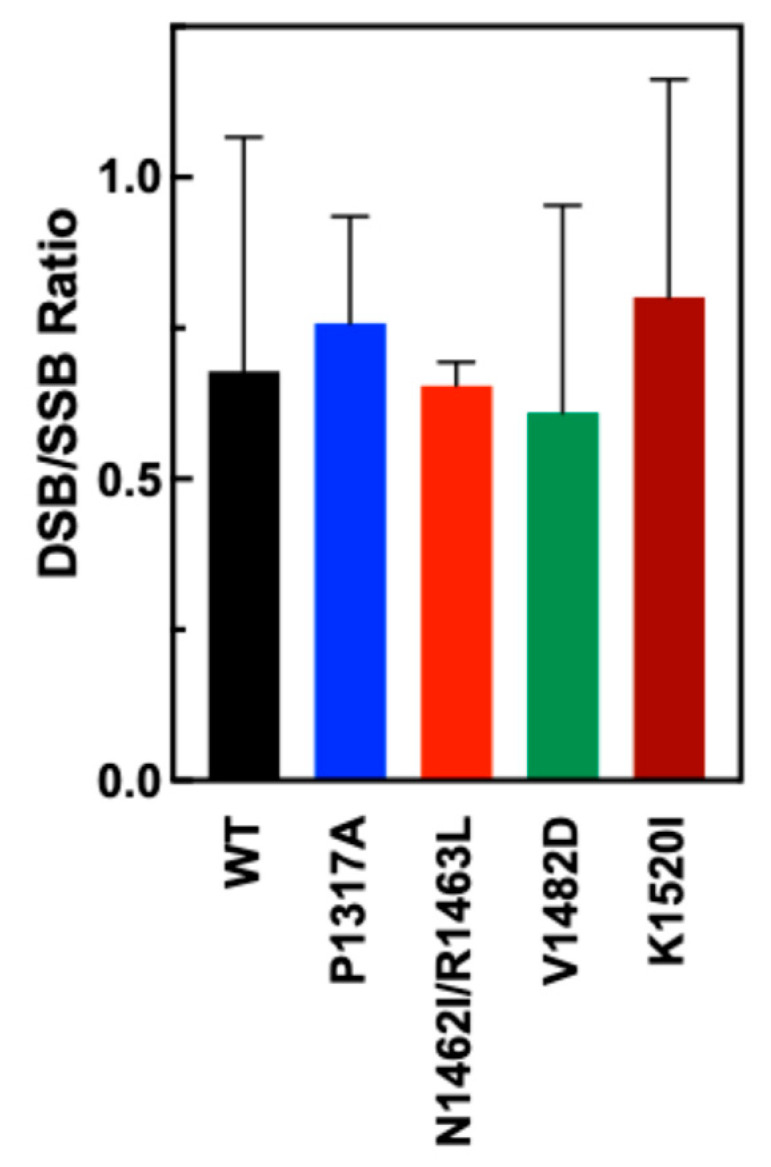
Ratio of double-strand breaks (linear) and single-strand breaks (nicked) in DNA when cleaved by WT and mutant TOP2A. DSB/SSB ratios were created by quantifying double-stranded breaks compared to single-strand breaks. A higher DSB/SSB ratio indicates increased coordination of the enzyme. Error bars show the standard deviation of at least four experiments.

To examine the DSB to SSB ratios for WT and CTD mutant TOP2A, nicked and DSB bands were quantified and a ratio was calculated based upon the quantification. As seen in Figure 5, the ratios for each of the mutants are comparable to the DSB/SSB ratio of WT, indicating no significant shifts in the coordination of the CTD mutants of TOP2A.

**Figure 6:**
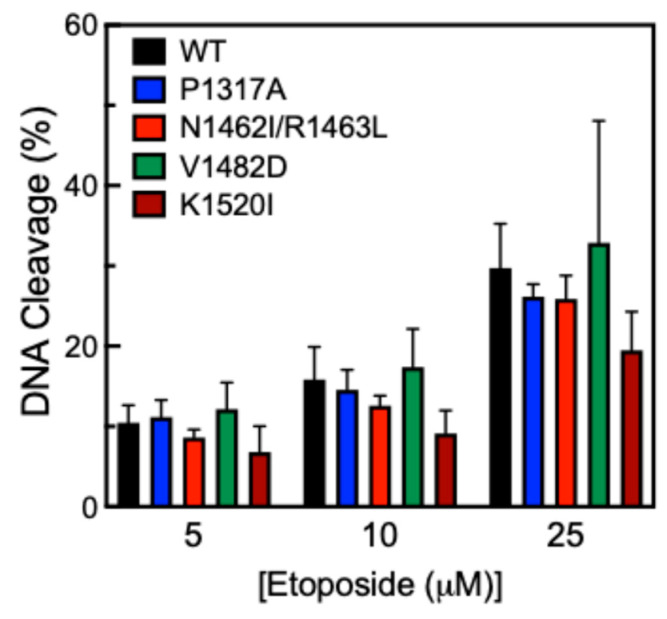
Etoposide titration cleavage of pHOT1 DNA by WT TOP2A and TOP2A mutants P1317A, N1462I/R1463L, V1482D, and K1520I. Etoposide concentration is represented in µM. Error bars indicate the standard deviation of at least four experiments.

Another way to measure DNA cleavage activity is to use the anticancer drug etoposide, which inhibits TOP2A from ligating cleaved DNA in the presence of the drug [40]. This mechanism is called “poisoning” and leads to an accumulation of DNA strand breaks. Etoposide acts in this manner and is referred to as a TOP2 poison. Cleavage assays were performed in the presence of 0–25 µM etoposide (Figure 6). While all CTD mutants were comparable to WT, K1520I shows a slight decrease in cleavage activity compared to WT, but this difference is not statistically significant.

### 2.3. Plasmid DNA Binding by CTD TOP2A Mutants

**Figure 7:**
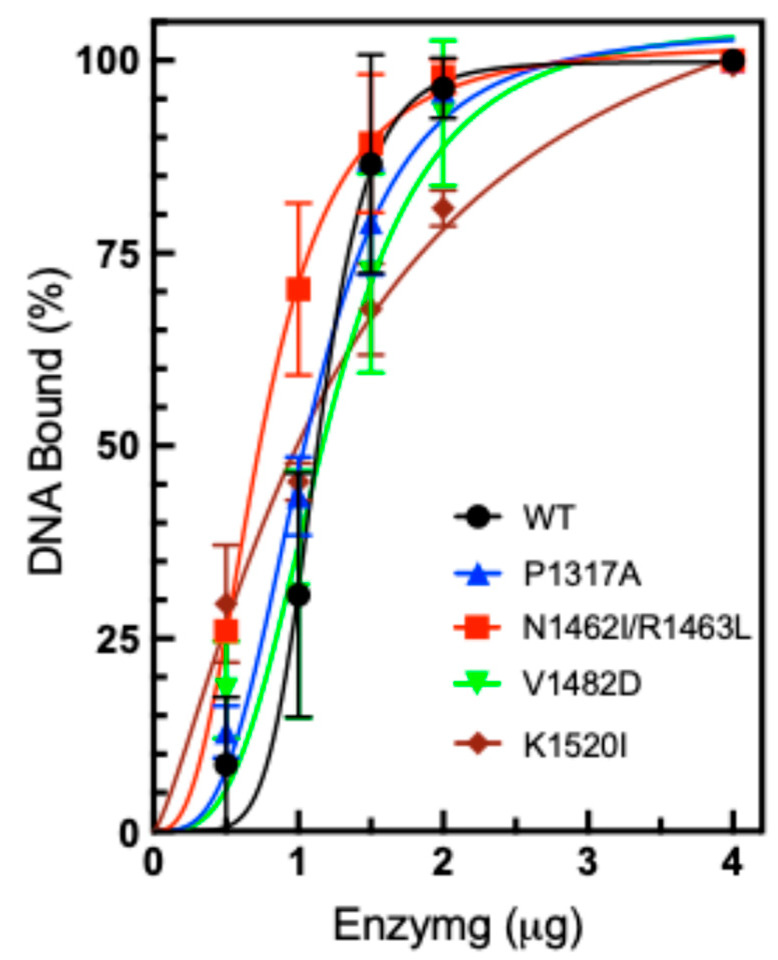
Plasmid DNA binding by TOP2A. Plasmid DNA was incubated in the presence of increasing concentrations of purified WT or mutant TOP2A, P1317A, N1462I/R1463L, V1482D, and K1520I (0.5–4 µg). Results are shown as a percentage of SC DNA remaining unshifted by binding to TOP2A. Error bars represent the standard deviation of three experiments.

While DNA cleavage activity does not appear to be significantly altered, DNA binding is also of interest to explore. It is possible that increased binding could lead to increased relaxation activity. In order to measure the level of DNA binding, we utilized the Electrophoretic Mobility Shift Assay (EMSA), which can be used to monitor enzyme-DNA binding [30,50]. Plasmid DNA was incubated with WT or mutant TOP2A and subsequently subjected to gel electrophoresis to separate the DNA molecules. The binding of DNA was assessed by observing the shifting of the supercoiled (SC) DNA band in the presence of increasing concentrations of WT or mutant TOP2A (Figure 7). In the presence of TOP2A, the plasmid DNA migrates more slowly due to its binding to the enzyme, and the degree of gel shift is proportional to the concentration of TOP2A. This assay was performed without the presence of Mg^2+^, which is necessary for cleavage and ligation, thereby confirming that the observed gel shift represents noncovalent interactions and not DNA cleavage products. Quantification of the gels revealed that most of the CTD mutants exhibited comparable DNA binding compared to WT TOP2A. The double mutant N1462I/R1463L TOP2A appears to bind more strongly, while the K1520I is slightly weaker compared to WT.

### 2.4. Kinetoplast DNA Decatenation by CTD TOP2A Mutants

While relaxation activity represents full catalytic function, TOP2 can also unlink DNA molecules. This activity is called decatenation, and it can be tested experimentally using kinetoplast DNA (kDNA) minicircles [30,51]. kDNA substrates represent multiple circles of DNA connected together, which cannot migrate into an agarose gel. In the presence of TOP2A and ATP, the enzyme can unlink the kDNA minicircles, resulting in smaller bands appearing in the agarose gel and the DNA in the wells gradually disappearing.

**Figure 8:**
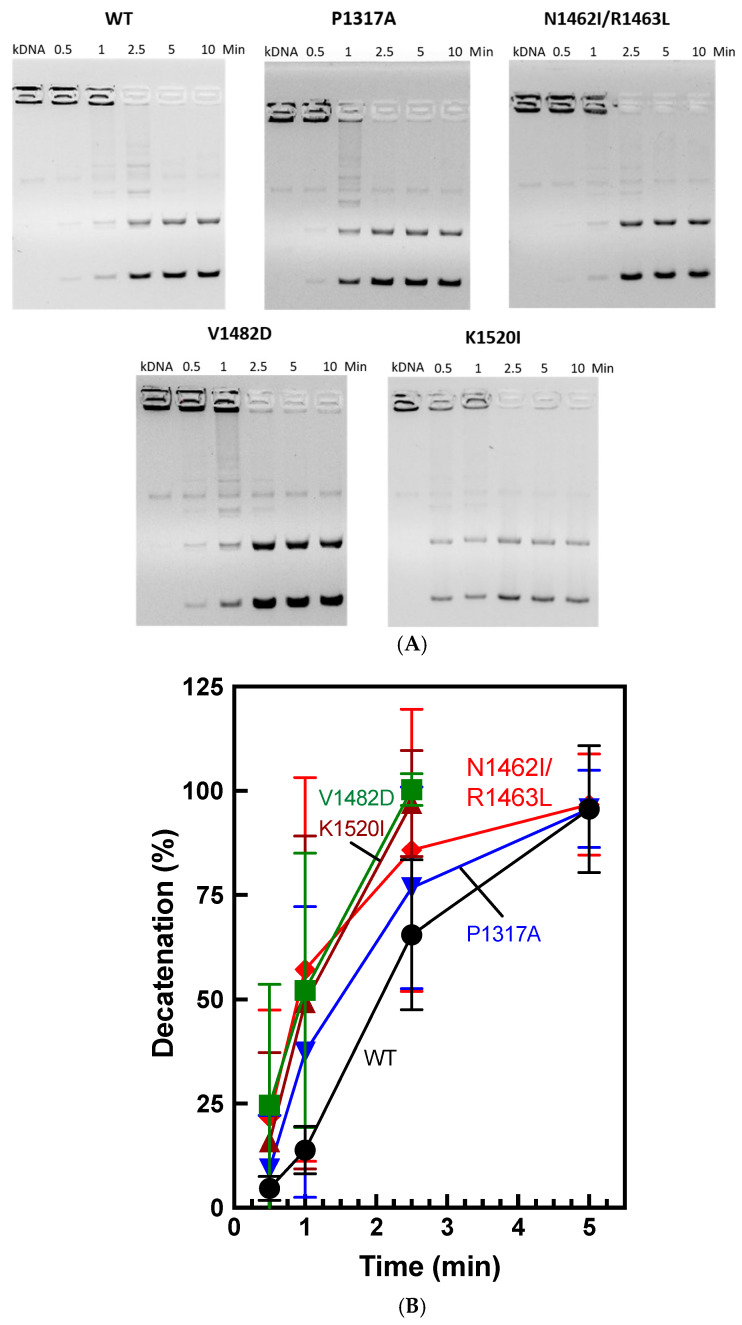
Decatenation of kinetoplast DNA (kDNA) using WT and mutant TOP2A. (**A**) Gel images show catenated (cat) kDNA in the absence of TOP2A (NT) as well as catenated kDNA as it is being decatenated (decat) at time points ranging from 0.5–10 min. Decatenation activity is shown for WT and mutants P1317A, N1462I/R1463L, V1482D, and K1520I. Gels are representative of four or more independent experiments. (**B**) Quantification of decatenated kDNA (lowest two bands) from 0.5–5 min. Error bars represent standard deviation of at least four experiments.

To test the ability of TOP2A mutants to decatenate DNA, we performed decatenation assays using kinetoplast DNA (kDNA) as a time course. As seen in Figure 8, WT enzyme decatenates the substrate fully by around 5 min. While all of the CTD mutants are also finished by ~2.5–5 min, K1520I and V1482D appear to be consistently faster than WT. N1462I/R1463L appears to be slightly faster than WT at the earliest time points.

### 2.5. N-Terminal Clamp Stability of TOP2A CTD Mutants

The ability of TOP2A to bind to and remain on DNA also involves the amino-terminus (N-terminus), which includes the ATPase domain and a region termed the N-terminal clamp [52,53]. This portion of the enzyme closes around the DNA after the two segments of DNA are captured. It is unclear whether the CTD impacts the function of the N-terminal clamp, but previous results from our lab did not show a major impact of CTD mutations on N-terminal clamp stability [46].

**Figure 9:**
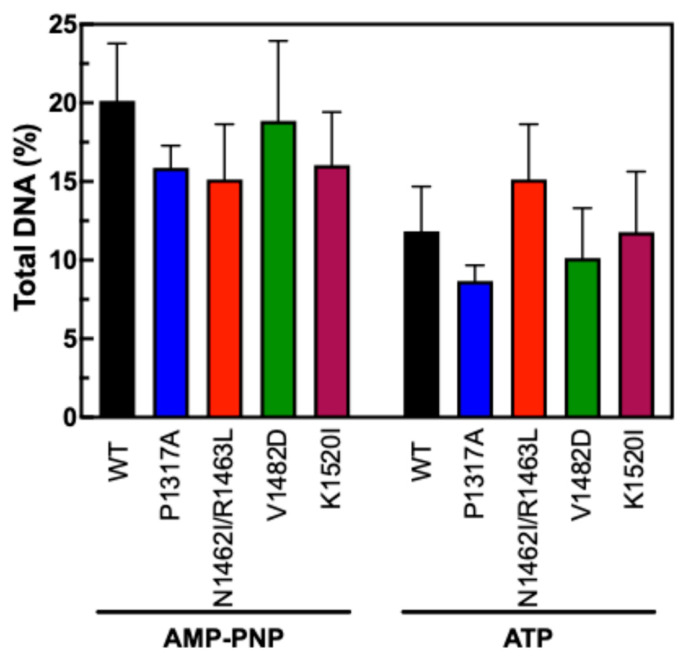
N-Terminal ATPase clamp stabilization. Results show the level of DNA bound to TOP2A enzymes following salt washes in the presence of AMP-PNP or ATP. Error bars represent the standard deviation of four experiments.

To measure the stability of the N-terminal clamp of TOP2A CTD mutants, we employed an updated fluorescence-based version of an assay that has been used in the past [46,54]. For this version, DNA and TOP2A were incubated together prior to being applied to a glass fiber filter that binds well to protein but not DNA. The filters were washed with low and high salt washes prior to being soaked in proteinase K to digest any remaining protein. If the DNA and enzyme are not bound, the DNA will flow through the filter. If the enzyme is clamped around the DNA, the DNA will remain on the filter until the proteinase K digestion. AMP-PNP is an ATP analog that is expected to “trap” the enzyme while ATP allows full catalytic activity. Our results in Figure 9 indicate that none of the CTD mutants significantly increased or decreased the stability of the N-terminal clamp. The AMP-PNP results show a slight increase above the ATP data, which is consistent with previous results indicating the ability of AMP-PNP to stabilize the clamp around DNA. Together, these results suggest that the effects seen on decatenation and relaxation are not due to any alteration of the function of the N-terminal clamp.

## 3. Discussion

The examination of the CTD of TOP2A has been challenging due to the disordered nature of the region. Structural biology has offered some insights along with enzymology and molecular biology. However, many questions remain unanswered about how the CTD functions and what role(s) it plays in regulating the function of TOP2A.

In this current study, we have extended our previous work on selective mutagenesis of the CTD by generating four new point mutants of TOP2A for analysis. These positions were initially selected based upon bioinformatic information, which turned out to be anomalous [31]. Nevertheless, the analysis of these mutants was completed.

In looking at overall catalytic activity, V1482D displays faster relaxation than the WT TOP2A. Interestingly, this does not appear to result from either increased DNA cleavage, increased coordination of DNA cleavage, or increased DNA binding as measured by EMSA.

Further, V1482D displays slightly faster decatenation than WT. In contrast, K1520I displays WT relaxation activity but increased decatenation compared to WT. Together, these results appear to indicate that relaxation and decatenation activities are not equivalent. This has been observed previously and implies that the CTD may impact one function without having an observable impact on the other [30].

The increase in relaxation or decatenation over WT levels may reflect a slight shift in the way the enzyme selects and interacts with substrates. TOP2A preferentially relaxes positive supercoils, and this selectivity is traced to the CTD [22,55]. Therefore, the observations here regarding V1482D and K1520I align with the fact that the CTD influences substrate selection. Without structural data on these sites and the nature of the interactions, it is difficult to clarify the nature of these interactions and how the changes impact the shape of the CTD and thus the interaction with DNA substrates.

None of the mutations impacted the N-terminal clamp stability in the presence of either AMP-PNP or ATP. This has been observed with previous CTD mutations as well [46]. This evidence may indicate that the role of the CTD does not regulate or significantly impact the ability of the N-terminus to capture DNA segments.

In terms of the bioinformatic clustering of the results via PSICalc, V1482D clusters with positions that caused decreased relaxation activity when mutated. Clearly, this region is important for interactions involved in relaxation. The opposite nature of the effect on the enzyme likely reflects the distinct nature of the mutations.

K1520I is part of the chromatin tether domain, which interacts with chromatin [25]. While the substrates used in these studies did not have histones, it is possible this region still interacts with DNA. The fact that decatenation occurs more quickly may reflect the ability to release the substrate more readily.

P1317A caused a decrease in relaxation activity without a major impact on decatenation. The double mutant N1462I/R1463L did not impact catalytic activities but did appear to slightly alter DNA binding. Each of these mutations is near regions that were mutated previously and impacted relaxation activity [30]. The impact of these mutations must be considered individually as influencing certain regions of the CTD.

**Figure 10:**
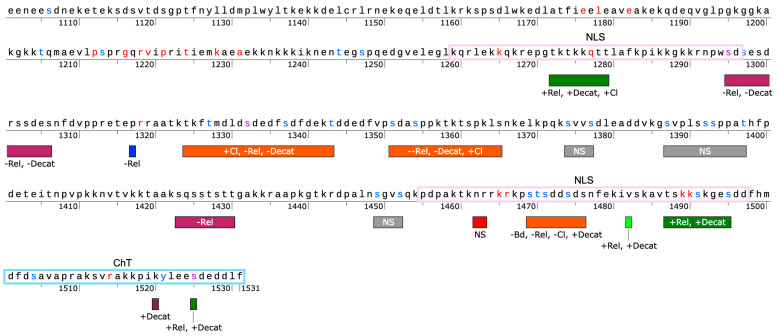
Map of TOP2A CTD mutation effects. The map above shows the region from amino acid 1100–1531 of TOP2A. Mutants from the current study are shown in blue (P1317A), red (N1462I/R1463L), bright green (V1482D), and maroon (K1520I). Mutants from a previous CTD study [30] are also mapped as regions in green (increased activity), purple (decreased activity), orange (mixed effects), or gray (no significant effects, NS). Activities are abbreviated as follows: + = increased activity, - = decreased activity, -- = strongly decreased activity; Rel = relaxation; Decat = decatenation; Cl = DNA cleavage; Bd = DNA binding; NS = no significant effects. Other features denoted include the Nuclear Localization Sequences (NLS, light purple box) and the Chromatin Tether Domain (ChT, teal box). Residues in red represent positions that are invariant among TOP2A [31], blue sites are known to be phosphorylated in association with mitosis [29,48], and purple represents sites that are both invariant and are known to be modified.

Taken together, these results support the fact that the CTD does impact TOP2A catalytic function but that not all positions have a discernable impact, at least with the amino acid substitutions that were selected. Further work needs to be done to explore the segregation of function between relaxation and decatenation. To aid in this work, Figure 10 provides a map of the CTD based upon this current work along with our previous work, indicating regions where activity levels increase or decrease following mutation [30,46]. As seen in the map, there appear to be regions that generally increase catalytic activity, such as ~1270–1280 and ~1485–1495. There also appear to be regions that decrease catalytic activity, such as ~1295–1306 and ~1423–1430. On the other hand, some regions appear to have a variable effect, such ~1323–1343 and ~1351–1365, but it should be noted that these two regions appear to have a more negative impact on overall catalytic activity.

## 4. Materials and Methods

### 4.1. Enzymes, Substrates, and Reagents

Enzymes were expressed and purified using a previously-designed construct for human TOP2A with an N-terminal domain 6x-His-tag sequence [30]. Mutagenesis and sequencing to validate changes were carried out by GenScript (Piscataway, NJ, USA). The amino acid changes are documented in Figure 1 and Supplemental Table S1. Sequencing data are available upon request. Wild-type and mutant human TOP2A were individually expressed in S. cerevisiae JEL1Δtop1 cells from the pESC-URA-TOP2A expression construct. Enzymes were purified as described previously [30]. The enzymes were stored at −80 °C as a 1 mg/mL (4 μM) stock in 50 mM Tris−HCl, pH 7.7, 0.1 mM ethylenediaminetetraacetic acid (EDTA), 750 mM KCl, and 5% glycerol. Example gel of purified proteins can be found in Supplementary Materials Figure S1.

Negatively supercoiled pHOT1 and pBR322 plasmid DNA were purified from Escherichia coli using a Plasmid Mega Kit (Qiagen, Hilden, Germany). Kinetoplast DNA (kDNA) was purchased from Topogen (Buena Vista, CO, USA). Etoposide (Sigma, St. Louis, MO, USA) was stored as a 20 mM stock in 100% DMSO at 4 °C. Etoposide was diluted to 2 mM in 10% DMSO for preparation of working solutions.

### 4.2. Topoisomerase IIα Plasmid DNA Relaxation Assay

Plasmid relaxation reactions were performed using a previously used procedure [30]. Each reaction mixture contained 14 nM WT or mutant TOP2A, 5 nM negatively supercoiled pBR322 plasmid DNA, and 1 mM ATP in 20 µL of 10 mM Tris−HCl, pH 7.9, 175 mM KCl, 0.1 mM Na_2_EDTA, 5 mM MgCl_2_, and 2.5% glycerol. Relaxation time courses of up to 30 min were started by the addition of enzyme and incubated at 37 °C for varying time points before stopping by adding 3 µL of stop solution [77.5 mM Na_2_EDTA, 0.77% sodium dodecyl sulfate (SDS)]. After stopping the reactions, the reaction was mixed with 2 µL of 5x nucleic acid sample loading buffer. Samples were loaded into a 1.33% agarose TBE gel and subjected to electrophoresis at 150 V for 2 h. The gel was stained in ethidium bromide and then visualized using a Bio-Rad ChemiDoc MP Imaging System and Image Lab Software version 6.1 (Hercules, CA, USA). DNA relaxation was monitored by the conversion of the supercoiled plasmid DNA to relaxed topoisomers.

### 4.3. Topoisomerase IIα Plasmid DNA Cleavage Assay

Plasmid DNA cleavage reactions were performed using an adapted procedure from Fortune and Osheroff [56]. Reaction mixtures contained 144 nM TOP2A, 5 nM plasmid DNA, 0–25 µM etoposide (1% DMSO final concentration) in a solution of 10 mM Tris (pH 7.9), 100 mM KCl, 0.1 mM Na_2_EDTA, 5 mM MgCl_2,_ and 2.5% glycerol. Each reaction is started by the addition of enzyme to the reaction mixture and incubated at 37 °C for 6 min prior to the addition of 2 µL 2.5% SDS to stop the reaction. The reactions are removed from heat and 2 µL 250 mM Na_2_EDTA and 2 µL 1x ProK in 50 mM Tris pH 7.9, 1 mM CaCl_2_ are added to each reaction. The reactions are incubated at 45 °C for 30 min, after which 2 μL Nucleic Acid Sample Loading Buffer (Bio-Rad, Hercules, CA, USA) is added to each tube. The reaction tubes are electrophoresed in a 1% TAE gel at 125 V for 2 h. Results of the experiment are visualized and quantified using a Bio-Rad ChemiDoc MP Imaging System and Image Lab Software version 6.1 (Bio-Rad, Hercules, CA, USA).

### 4.4. Topoisomerase IIα Binding of Plasmid DNA

Plasmid DNA binding reactions were performed using a previously used procedure [30]. The reaction mixtures consist of approximately 0–4 μg (0–576 nM) of wild-type or mutant TOP2A and 0.3 μg pHOT-1 DNA plasmid in a 20 μL volume of 10 mM Tris−HCl, pH 7.9, 150 mM KCl, 1 mM EDTA, 5 mM MgCl_2_, and 2.5% glycerol. Following the addition of the enzyme, the reaction mixture is incubated at 37 °C for 6 min to allow for binding. The binding reactions are then mixed with 2 μL Nucleic Acid Sample Loading Buffer (Bio-Rad, Hercules, CA, USA), and the samples are then electrophoresed in 2% TAE gels containing ethidium bromide at 150 V for 2 h. DNA bands are visualized and quantified using a Bio-Rad ChemiDoc MP Imaging System and Image Lab Software (Bio-Rad, Hercules, CA, USA).

### 4.5. Decatenation of Kinetoplast DNA (kDNA) by Topoisomerase IIα

The kDNA decatenation assays were performed as previously described with some modifications [30]. Each reaction mixture contained 14 nM TOP2A enzyme, 0.21 μg of kDNA, and 1 mM ATP in a 20 μL volume composed of 10 mM Tris−HCl, pH 7.9, 175 mM KCl, 5 mM MgCl_2_, 0.1 mM Na_2_EDTA, and 2.5% glycerol. After the addition of the enzyme, the mixture is incubated at 37 °C and stopped using 3 μL of a stop solution composed of 0.5% SDS and 77 mM Na_2_EDTA at time points ranging from 0.5–15 min. Each sample was then treated with proteinase K and incubated at 45 °C for 30 min. Nucleic Acid Sample Loading Buffer (Bio-Rad, Hercules, CA, USA) was then added to the samples before being electrophoresed in 1% TBE gels for 60 min at 150 V. The gels were stained with ethidium bromide before being imaged using a Bio-Rad ChemiDoc MP Imaging System and Image Lab Software (Bio-Rad, Hercules, CA, USA).

### 4.6. Clamp Closing

The clamp closing assays were performed using an adapted procedure from Roca and Wang [57]. All reaction mixtures were incubated at 37 °C for 20 min and contained 0.5 μg pBR322 plasmid DNA in a solution of clamp closing buffer: 50 mM Tris (pH 7.7), 100 mM KCl, 1 mM Na_2_EDTA, and 8 mM MgCl_2_. Individual reaction conditions contained 0.3 μg WT or mutant TOP2A, 1 mM ATP, or AMP-PNP. Glass fiber filters were prepared by soaking the filters for 20 min with 60 μL of SSDNA pre-wash, containing 6 μg acetylated BSA, 6 μg salmon sperm DNA, 50 mM Tris (pH 7.7), 100 mM KCl, 1 mM Na_2_EDTA, and 8 mM MgCl_2_, which were then washed to remove the excess salmon sperm DNA. The reactions were placed on the filters then run through a series of washes with 200 μL of low salt wash (50 mM Tris [pH 7.7], 100 mM KCl, 1 mM Na_2_EDTA, and 8 mM MgCl_2_), 300 μL of high salt wash (1 M NaCl, 50 mM Tris [pH 7.7], 100 mM KCl, 1 mM Na_2_EDTA, and 8 mM MgCl_2_), and 20 μL of proteinase K wash (2 μL of 1 mg/mL proteinase K in 18 μL of low salt wash). All washes were centrifuged at 1000 rpm for 60 s. The proteinase K wash was incubated at 45 °C for 20 min. The flow-through was collected and the DNA from the low salt wash and proteinase K wash was directly quantified. The high salt wash flow-through was purified using a Monarch Plasmid DNA Purification Kit before being quantified. DNA samples were quantified using PreciseGreen, a dsDNA-specific dye, on an Agilent BioTek Synergy H1 Multi-Mode Microplate Reader and Gen6 Data Analysis Software (Agilent, Santa Clara, CA, USA).

### 4.7. Data Visualization

All graphs presented in this study were generated using Graphpad Prism 10 (Graphpad Software, Boston, MA, USA).

## 5. Conclusions

This work lays the foundation for additional research on the CTD. It appears that this work, consistent with previous research, supports the idea that the CTD influences TOP2A catalytic activity, especially as it relates to relaxation and decatenation. This result is likely broadly applicable to TOP2A. More research remains to further chart the impacts of mutations on the TOP2A CTD structure and function.

## Data Availability

Datasets are available upon reasonable request to the corresponding author.

## Supplementary Materials

The following supporting information can be downloaded at: https://www.mdpi.com/article/10.3390/ijms26083604/s1.

## Abbreviations

The following abbreviations are used in this manuscript:

BSA	Bovine serum albumin
ChT	Chromatin tether domain
CTD	C-terminal domain
decat	Decatenated
DSB	Double strand break
EMSA	Electrophoretic mobility shift assay
IDP	Intrinsically disordered proteins
IDR	Intrinsically disordered protein regions
kDNA	Kinetoplast DNA
TOP2	Topoisomerase II
TOP2A	Topoisomerase IIα
TOP2B	Topoisomerase IIβ
NLS	Nuclear localization sequence
NT	No topoisomerase II (without the presence of TOP2A)
N-terminus	Amino-terminus
Rel	Relaxed
SC	Supercoiled
SSB	Single strand break
WT	Wild-type
