# Supplementary material for: Mutagenesis of Intrinsically Disordered Domain Impacts Topoisomerase IIα Catalytic Activity
Source: Int J Mol Sci. 2025 Apr 11;26(8):3604. doi: 10.3390/ijms26083604 (PMC12026768; doi:10.3390/ijms26083604)
Supplement: Supplementary file 1 [file ijms-26-03604-s001.zip › ijms-3545274-supplementary.pdf]

Supplemental Table S1. TOP2A Mutant Sites and Corrected Cluster Information

Mutant	PSICalc Clustered Positions	Additional Information	Notes
P1317A	183, 189, 295, 1316, 1317, 1358, 1369, 1382, 1488, 1512	10th order cluster* primarily CTD with some ATPase positions	Near invariant position (R1318); previous mutations on either side of this position reduced relaxation
N1462I	1462: large cluster	19th Order cluster with positions across various domains	Within the second NLS; near a previously mutated region that significantly reduced relaxation
R1463L	1256, 1349, 1354, 1460, 1463	Exclusively CTD Cluster	
V1482D	453, 812, 1075, 1235, 1301, 1398, 1400, 1401, 1482, 1502	10th order cluster that includes primarily CTD residues (begins with 1301 and 1075)	Within the second NLS; near a previously mutated region that significantly reduced relaxation
K1520I	Very large cluster	Only clusters near end of clustering process and groups with hundreds of positions	Within the ChT domain known to bind histones; charged position that may be involved in DNA binding

*PSICalc identifies clusters of amino acids and these can be grouped by how many amino acids are part of the cluster. A 10th order cluster has 10 amino acids where a pairwise cluster would only have two. As PSICalc proceeds through rounds of cluster identification, smaller clusters can grow as additional amino acids or clusters can be grouped with clusters already identified. For more information see ref. [1].

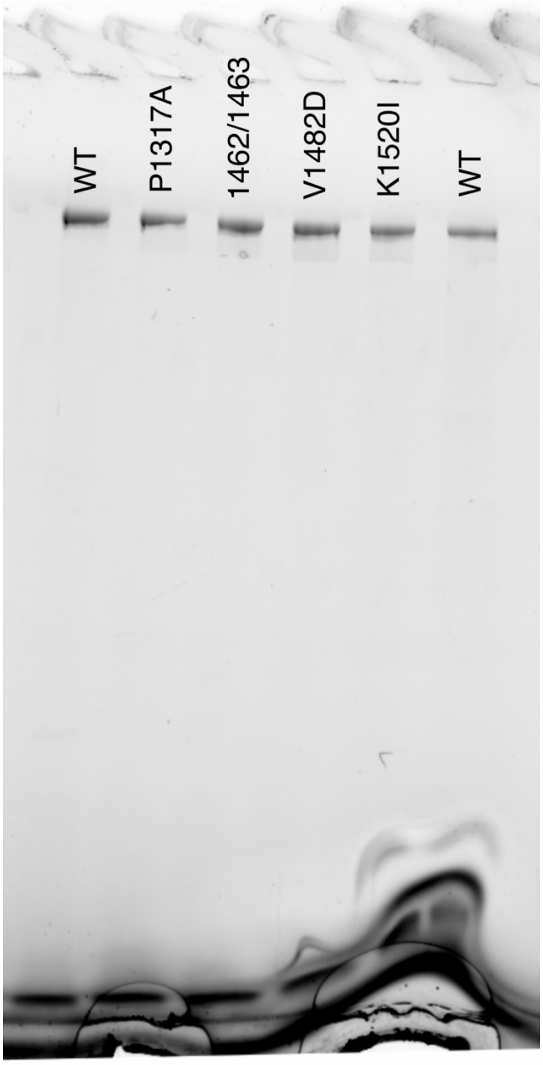

Figure S1: Protein Gel of WT TOP2A and TOP2A Mutants. Bio-Rad Stain Free Any-KD SDS-PAGE gel with 1 µg samples from WT, P1317A, N1462I/R1463L, V1482D, and K1520I mixed with Laemmli buffer with beta-mercaptoethanol and heated for 5 min at 50°C. Gel run at 300 V for 20 min. Stain Free gel dye responds to protein in the gel. Bands at the very bottom represent dye front.

References:

1. Townsley, T.D.; Wilson, J.T.; Akers, H.; Bryant, T.; Cordova, S.; Wallace, T.L.; Durston, K.K.; Deweese, J.E. PSICalc: a novel approach to identifying and ranking critical non-proximal interdependencies within the overall protein structure. *Bioinform Adv* **2022**, *2*, vbac058, doi:10.1093/bioadv/vbac058.
